# Disability-adjusted life years and mortality rate attributed to unsafe sex and drug use for AIDS in the Middle East and North Africa countries

**DOI:** 10.1186/s13690-020-00511-1

**Published:** 2020-12-09

**Authors:** Farid Najafi, Fatemeh Khosravi, Mojtaba Ghalandari, Mitra Darbandi

**Affiliations:** 1 Research Center for Environmental Determinants of Health (RCEDH), Health Institute, Kermanshah University of Medical Sciences, Kermanshah, Iran; 2 Cardiovascular Research Center, Kermanshah University of Medical Sciences, Kermanshah, Iran; 3 Mahneshan Health Center, Zanjan University of Medical Sciences, Zanjan, Iran; 4 Department Epidemiology, Public Health College, Kermanshah University of Medical Sciences, Kermanshah, Iran

**Keywords:** Global burden of disease, Mortality, Unsafe sex, Human immunodeficiency virus

## Abstract

**Background:**

The Middle East and North Africa, is one of few regions where the number of new human immunodeficiency virus infections is increasing. The present study aimed to estimate the attributable burden of unsafe sex and drug use in Acquired immunodeficiency syndrome in the Middle East and North Africa countries.

**Methods:**

We used the Global Burden of Disease data 2017 to estimate the attributable mortality and disability-adjusted life-years to unsafe sex and drug use in Acquired immunodeficiency syndrome in the Middle East and North Africa countries (21 countries) from 1990 to 2017 by region, sex and age. The percent change was calculated at three time points by country and sex.

**Results:**

The rate of Disability-adjusted life years/100,000 attributed to drug use for Acquired immunodeficiency syndrome increased 1.10 (95% CI: 0.75–1.71) to 13.39 (95% CI: 9.98–18.17) in women of Middle East and North Africa countries from 1990 to 2017, and there is an increasing trend in Disability-adjusted life years attributable to drug use for Acquired immunodeficiency syndrome in men. The rate of Disability-adjusted life **years**/100,000 attributed to unsafe sex for Acquired immunodeficiency syndrome increased in women of Middle East and North Africa countries, 5.15 (95% CI: 3.34–8.07) to 53.44 (95% CI: 38.79–75.89); and 10.06 (95% CI: 6.61–16.18) to 46.16 (95% CI: 31.30–72.66) in men. Age-standardized mortality rate attributed to drug use and unsafe sex for Acquired immunodeficiency syndrome increased from 1990 to 2017 in both sex in Middle East and North Africa countries.

**Conclusion:**

The rate of Disability-adjusted life years /100,000 and age-standardized mortality rate attributed to unsafe sex and drug use increased in Middle East and North Africa from 1990 to 2017. While most of such countries have traditional cultures with religious believes, such increase need to be addressed in more depth by all policy makers.

## Background

The Middle East and North Africa (MENA), is one of few regions where the number of new human immunodeficiency virus (HIV) infections is increasing [1]. New HIV infections in the MENA region have increased by 31% since 2001, which is the highest increase among all regions in the world [2]. Between 2000 and 2015, the increase in the number of new infections was estimated at over a third, while that of Acquired immunodeficiency syndrome (AIDS) -related deaths, at over threefold [3, 4]. However, the current prevalence of 0.1% is still among the lowest rates globally [2]. The majority of these infections seem to be occurring among key populations, including people who inject drugs (PWID), their sexual partners and sex workers (SWs). Studies have shown that transmission through unsafe sex and drug users is high and significant, and these are two important risk factors for HIV/AIDS [1, 5–7].

To reduce the burden of HIV/AIDS, it is important to identify and estimate the attributable burden of risk factors associated with HIV/AIDS. Globally, in 2013, more than 10 million disability-adjusted life-years (DALYs) were estimated to be attributable to previous exposure to HIV, Hepatit B virus (HBV), and Hepatit C virus (HCV) via injecting drug use (IDU). This represents a four-times increase in DALYs since 1990 [7]. Injection drug use increases the risk of occurrence and AIDS-related deaths [8, 9]. The reasons for this are likely multifactorial and not entirely clear, but may be related to lifestyle behaviors more common in this population that are generally detrimental to overall heath (e.g. alcohol and illicit substance abuse, tobacco smoking) [10, 11]. HIV continues to spread through sexual transmission worldwide. Studies indicate that a significant number of HIV positive individuals engage in high-risk sexual practices. Though many persons living with HIV/AIDS either abstain from sex or significantly reduce risky sexual behavior, a significant percentage of HIV positive persons (ranging from 10% to as high as 64%) continue to engage in risky sexual behaviors [12–16]. Many of people with HIV continue to engage in unprotected sexual behaviors that place others at risk for infection and place themselves at risk for contracting secondary infections (e.g., syphilis, gonorrhea, herpesvirus-6) that may accelerate HIV disease [17, 18].

Recognizing the attributable burden of risk factors will support the policy making for prevention, and control of HIV/AIDS. Drug use and unsafe sex are two major risk factors known to control that can reduce the burden of HIV/AIDS [7, 19]. The aim of study was to estimate the attributable burden of unsafe sex and drug use in AIDS in the MENA countries based on findings from the Global Burden of Disease (GBD) Study from 1990 to 2017.

## Methods

The GBD study series provides comprehensive global information about diseases and risk factors. This information is based on geographical areas investigating the incidence, prevalence and mortality, as well as disease burden attributable to risk factors by age and sex over time. The GBD data in 2017 was employed in this study. The data on region, age, and sex is found on Institute for Health Metrics and Evaluation (IHME) reports (https://vizhub.healthdata.org/gbd-compare/). We extracted the data for the MENA countries, which included 21 countries **(**Afghanistan, Algeria**,** Bahrain, Egypt, Iran, Iraq, Jordan, Kuwait, Lebanon, Libya, Morocco, Oman, Palestine, Qatar, Saudi Arabia, Sudan, Syria, Tunisia, Turkey, United Arab Emirates, Yemen), by 1990, 2007 and 2017.

After cleaning the data, attributable mortality and DALYs to drug use and unsafe sex for AIDS was evaluated. The attributable number of DALY and death of the risk factors were estimated by multiplying DALYs/mortality rate from HIV/AIDS by the population attributable fraction for HIV/AIDS due to that risk factor.

After extracting the data, we reported attributable burden of drug use and unsafe sex in AIDS the information separately for men and women in the MENA countries by two Tables 1 and 2. The percent change was calculated at three time points by country and sex. We report age-standardized estimates, and 95% confidence intervals (CI) for rates or numbers of DALYs or mortality. We used radar figures to show trend of the rate of DALYs/100,000 and age-standardized mortality rate attributed to drug use and unsafe sex in AIDS at MENA countries in men and women. We used stack figures to show age trend of the attributable burden of unsafe sex and drug use in AIDS at MENA countries. Analysis was done using STATA software version 14.2 (StataCorp, College Station, TX, USA) and designing of radar and stack figures in R software.

**Table 1:** Attributable mortality and burden of drug use in AIDS at MENA countries

Country	sex	DALY (Per 100,000 population)			Percent change (%)			Age standardized Mortality Rate (Per 100,000 population)			Percent change (%)		
		1990	2007	2017	1990–2007	2007–2017	1990–2017	1990	2007	2017	1990–2007	2007–2017	1990–2017
Afghanistan	F	1.30 (0.06–4.85)	3.15 (3.15–11.99)	3.57 (0.13–16.82)	142.31	13.33	174.62	0.02 (0.00–0.09)	0.06 (0.00–0.24)	0.06 (0.00–0.35)	200.00	0.00	200.00
	M	3.81 (0.31–14.18)	7.69 (0.98–30.27)	9.61 (0.28–59.05)	101.84	24.97	152.23	0.08 (0.30–0.31)	0.16 (0.01–0.66)	0.20 (0.27–1.00)	100.00	25.00	150.00
Algeria	F	1.11 (0.26–5.57)	5.28 (5.28–25.23)	4.13 (0.44–29.77)	375.68	−21.78	272.07	0.02 (0.00–0.11)	0.10 (0.01–0.51)	0.07 (0.64–0.00)	400.00	−30.00	250.00
	M	1.46 (0.21–7.35)	5.71 (0.68–24.19)	5.75 (0.43–25.83)	291.10	0.70	293.84	0.03 (0.00–0.16)	0.12 (0.00–0.56)	0.12 (0.00–0.58)	300.00	0.00	300.00
Bahrain	F	4.56 (3.52–5.41)	12.63 (12.63–15.15)	10.90 (8.54–13.55)	176.97	−13.70	139.04	0.09 (0.07–0.11)	0.26 (0.20–0.32)	0.22 (0.17–0.28)	188.89	− 15.38	144.44
	M	34.58 (27.1–40.72)	37.31 (34.89–40)	16.56 (14.36–19.58)	7.89	−55.62	− 52.11	0.81 (0.65–0.94)	0.96 (1.89–0.03)	0.41 (0.35–0.48)	18.52	−57.29	−49.38
Egypt	F	0.40 (0.21–0.58)	0.74 (0.74–0.93)	0.30 (0.17–0.59)	85.00	−59.46	−25.00	0.00 (0.00–0.01)	0.01 (0.01–0.01)	0.00 (0.00–0.01)	–	−100.00	–
	M	0.84 (0.41–1.16)	0.93 (0.69–1.27)	0.63 (0.41–0.96)	10.71	− 32.26	− 25.00	0.01 (0.01–0.02)	0.01 (0.01–0.02)	0.01 (0.00–0.01)	0.00	0.00	0.00
Iran	F	1.27 (1.05–1.47)	8.47 (8.47–9.02)	20.62 (17.31–24.01)	566.93	143.45	1523.62	0.02 ((0.02–0.03)	0.18 (0.17–0.19)	0.47 (0.38–0.55)	800.00	161.11	2250.00
	M	5.59 (4.51–6.53)	27.53 (25.92–29)	33.52 (30.17–37.5)	392.49	21.76	499.64	0.11 (0.08–0.13)	0.56 (0.53–0.58)	0.69 (0.62–0.78)	409.09	23.21	527.27
Iraq	F	0.24 (0.14–0.37)	1.24 (1.24–1.59)	1.56 (1.07–2.18)	416.67	25.81	550.00	0.00 (0.00–0.00)	0.02 (0.01 0.03)	0.02 (0.02–0.04)	–	0.00	–
	M	0.2 (0.11–0.31)	1.04 (0.81–1.35)	1.24 (0.87–1.69)	420.00	19.23	520.00	0.00 (0.00–0.00)	0.02 (0.01–0.02)	0.02 (0.01–0.03)	–	0.00	–
Jordan	F	0.30 (0.20–0.42)	0.79 (0.79–1.03)	0.58 (0.42–0.78)	163.33	−26.58	93.33	0.00 (0.00–0.00)	0.01 (0.01 0.02)	0.01 (0.00–0.01)	–	0.00	–
	M	0.13 (0.01–0.18)	0.54 (0.41–0.7)	0.93 (0.48–1.46)	315.38	72.22	615.38	0.00 (0.00–0.00)	0.01 (0.00–0.01)	0.01 (0.00–0.03)	–	0.00	–
Kuwait	F	0.77 (0.57–1.01)	1.34 (1.34–1.78)	0.62 (0.46–0.83)	74.03	−53.73	−19.48	0.01 (0.01–0.02)	0.02 (0.01–0.03)	0.01 (0.00–0.01)	100.00	−50.00	0.00
	M	1.36 (1.01–1.68)	0.64 (0.5–0.79)	0.56 (0.44–0.7)	−52.94	−12.50	− 58.82	0.02 (0.02–0.03)	0.01 (0.00–0.01)	0.01 (0.00–0.01)	− 50.00	0.00	− 50.00
Lebanon	F	5.36 (0.24–17.71)	6.33 (6.33–27.74)	5.96 (0.21–27.15)	18.10	−5.85	11.19	0.10 (0.00–0.34)	0.12 (0.00–0.55)	0.11 (0.00–0.55)	20.00	−8.33	10.00
	M	15.44 (1.51–48.3)	11.75 (0.94–58.33)	8.45 (0.49–57.39)	− 23.90	− 28.09	−45.27	0.33 (0.02–1.09)	0.25 (0.01–1.25)	0.17 (0.01–1.25)	− 24.24	−32.00	−48.48
Libya	F	1.09 (0.07–3.83)	5.94 (5.94–23.02)	8.28 (0.89–31.37)	444.95	39.39	659.63	0.02 (0.00–0.07)	0.11 (0.01–0.46)	0.16 (0.01–0.64)	450.00	45.45	700.00
	M	1.47 (0.11–4.91)	6.64 (0.85–26.56)	9.13 (0.96–45.13)	351.70	37.50	521.09	0.03 (0.00–0.11)	0.14 (0.01–0.59)	0.19 (0.01–1.02)	366.67	35.71	533.33
Morocco	F	2.00 (0.13–6.38)	13.72 (13.72–61.9)	7.70 (0.48–44.44)	586.00	− 43.88	285.00	0.03 (0.00–0.13)	0.26 (0.00–1.22)	0.14 (0.00–0.96)	766.67	− 46.15	366.67
	M	2.91 (0.31–9.54)	16.31 (1.12–74.51)	11.48 (0.57–83.15)	460.48	−29.61	294.50	0.06 (0.00–0.21)	0.34 (0.01–1.66)	0.23 (0.00–1.83)	466.67	−32.35	283.33
Oman	F	0.58 (0.42–0.79)	2.25 (2.25–3.11)	3.14 (1.59–5.57)	287.93	39.56	441.38	0.01 (0.00–0.01)	0.04 (0.02–0.06)	0.06 (0.02–0.11)	300.00	50.00	500.00
	M	1.38 (1.01–1.75)	6.21 (4.47–8.28)	15.43 (7.56–24.01)	350.00	148.47	1018.12	0.03 (0.02–0.03)	0.13 (0.09–0.17)	0.35 (0.16–0.55)	333.33	169.23	1066.67
Palestine	F	0.07 (0.05–0.09)	0.95 (0.95–1.21)	1.30 (0.99–1.71)	1257.14	36.84	1757.14	0.00 (0.00–0.00)	0.01 (0.01–0.02)	0.02 (0.01–0.03)	–	100.00	–
	M	0.56 (0.31–0.78)	1.74 (1.36–2.18)	1.7 (1.3–2.2)	210.71	−2.30	203.57	0.01 (0.00–0.01)	0.03 (0.02–0.04)	0.03 (0.02–0.04)	200.00	0.00	200.00
Qatar	F	3.59 (2.47–4.89)	2.26 (2.26–3.18)	1.87 (1.24–2.58)	−37.05	−17.26	−47.91	0.06 (0.04–0.09)	0.04 (0.03–0.06)	0.03 (0.02–0.05)	−33.33	− 25.00	−50.00
	M	3.45 (2.41–4.66)	1.94 (1.47–2.49)	1.14 (0.88–1.47)	− 43.77	− 41.24	−66.96	0.07 (0.04–0.09)	0.04 (0.03–0.05)	0.02 (0.01–0.03)	−42.86	−50.00	− 71.43
Saudi Arabia	F	2.74 (1.65–4.38)	10.44 (10.44–13.17)	10.19 (7.2–13.85)	281.02	−2.39	271.90	0.05 (0.03–0.08)	0.21 (0.02–0.16)	0.20 (0.14–0.28)	320.00	−4.76	300.00
	M	3.15 (1.91–5.02)	8.82 (6.96–11.01)	7.69 (5.4–10.21)	180.00	−12.81	144.13	0.06 (0.04–0.10)	0.19 (0.15–0.23)	0.16 (0.11–0.21)	216.67	−15.79	166.67
Sudan	F	5.11 (2.94–10.43)	122.92 (122.92–186.15)	138.22 (90.81–201.91)	2305.48	12.45	2604.89	0.09 (0.05–0.19)	2.44 (1.56–3.62)	2.77 (1.84–4.00)	2611.11	13.52	2977.78
	M	11.78 (6.71–24.04)	106.92 (68.56–162.06)	98.4 (64.05–154.16)	807.64	−7.97	735.31	0.22 (0.12–0.47)	2.19 (1.42–3.30)	2.02 (1.30–3.16)	895.45	− 7.76	818.18
Syria	F	0.30 (0.21–0.42)	0.57 (0.57–0.80)	0.49 (0.31–0.80)	90.00	−14.04	63.33	0.00 (0.00–0.00)	0.01 (0.00–0.01)	0.00 (0.00–0.01)	–	−100.00	–
	M	0.14 (0.11–0.19)	0.22 (0.14–0.32)	0.14 (0.08–0.28)	57.14	−36.36	0.00	0.00 (0.00–0.00)	0.00 (0.00–0.00)	0.00 (0.00–0.00)	–	–	–
Tunisia	F	0.17 (0.04–0.83)	1.67 (1.67–8.30)	3.74 (0.17–17.74)	882.35	123.95	2100.00	0.00 (0.00–0.01)	0.03 (0.00–0.16)	0.07 (0.00–0.35)	–	133.33	–
	M	2.06 (0.31–9.65)	10.45 (0.99–49.85)	15.43 (0.78–58.27)	407.28	47.66	649.03	0.04 (0.00–0.20)	0.02 (0.00–1.09)	0.32 (0.00 1.29)	−50.00	1500.00	700.00
Turkey	F	0.01 (0–00.020)	0.07 (0.07–0.11)	0.19 (0.13–0.25)	600.00	171.43	1800.00	0.00 (0.00–0.00)	0.00 (0.00–0.00)	0.00 (0.00–0.00)	–	–	–
	M	0.02 (1–0.04)	0.16 (0.11–0.25)	0.29 (0.2–0.4)	700.00	81.25	1350.00	0.00 (0.00–0.00)	0.00 (0.00–0.00)	0.00 (0.00–0.00)	–	–	–
United Arab Emirates	F	0.59 (0.03–2.00)	3.78 (3.78–14.14)	4.63 (0.50–17.58)	540.68	22.49	684.75	0.01 (0.00–0.04)	0.07 (0.00–0.27)	0.09 (0.00–0.35)	600.00	28.57	800.00
	M	1.19 (0.11–3.97)	6.78 (0.9–30.05)	25.2 (1.02–146.01)	469.75	271.68	2017.65	0.02 (0.00–0.09)	0.14 (0.01–0.64)	1.05 (0.01–6.89)	600.00	650.00	5150.00
Yemen	F	0.45 (0.02–1.92)	0.98 (0.98–3.97)	0.91 (0.04–4.86)	117.78	−7.14	102.22	0.00 (0.00–0.04)	0.01 (0.00–0.07)	0.01 (0.00–0.09)	–	0.00	–
	M	1.05 (0.01–4.84)	1.84 (0.2–7.36)	1.86 (0.07–11.54)	75.24	1.09	77.14	0.02 (0.00–0.11)	0.03 (0.00–0.15)	0.03 (0.00–0.25)	50.00	0.00	50.00
North Africa and Middle East	**F**	**1.10 (0.75–1.71)**	**10.60 (10.6–15.27)**	**13.39 (9.98–18.17)**	863.64	26.32	1117.27	**0.02 (0.01–0.03)**	**0.21 (0.14–0.30)**	**0.27 (0.20–0.36)**	950.00	28.57	1250.00
	**M**	**2.63 (1.91–3.64)**	**13 (9.86–17.82)**	**13.17 (10.26–17.83)**	394.30	1.31	400.76	**0.05 (0.03–0.07)**	**0.26 (0.20–0.37)**	**0.27 (0.21–0.37)**	420.00	3.85	440.00

**Table 2:** Attributable mortality and burden of unsafe sex in AIDS at MENA countries

Country	sex	DALY (Per 100,000 population)			Percent change (%)			Age standardized Mortality Rate (Per 100,000 population)			Percent change (%)		
		1990	2007	2017	1990–2007	2007–2017	1990–2017	1990	2007	2017	1990–2007	2007–2017	1990–2017
Afghanistan	F	6.66 (0.33–24.88)	16.55 (1.93–63.63)	20.05 (0.74–98.52)	148.50	21.15	201.05	0.13 (0.00–0.50)	0.34 (0.02–1.30)	038 (0.00–2.06)	161.54	11.76	192.31
	M	19.24 (1.70–75.13)	39.51 (5.13–153.41)	51.16 (1.70–282.96)	105.35	29.49	165.90	0.44 (0.02–1.84)	0.90 (0.08–3.73)	1.12 (0.02–6.53)	104.55	24.44	154.55
Algeria	F	5.52 (1.29–28.55)	26.94 (4.46–129.03)	21.54 (2.53–149.86)	388.04	−20.04	290.22	0.11 (0.02–0.59)	0.53 (0.06–2.73)	0.41 (0.02–3.30)	381.82	−22.64	272.73
	M	7.43 (1.10–38.11)	30.07 (3.79–126.19)	31.38 (2.69–130.65)	304.71	4.36	322.34	0.16 (0.01–0.91)	0.69 (0.04–3.04)	0.73 (0.01–3.45)	331.25	5.80	356.25
Bahrain	F	3.31 (2.58–3.95)	9.04 (7.31–11.02)	8.32 (6.53–10.12)	173.11	−7.96	151.36	0.07 (0.06–0.09)	0.20 (0.15–0.25)	0.18 (0.14–0.22)	185.71	−10.00	157.14
	M	24.31 (19.55–28.59)	28.57 (30.98–26.47)	13.82 (11.94–15.88)	17.52	−51.63	−43.15	0.62 (0.50–0.72)	0.81 (0.75–0.87)	0.37 (0.32–0.42)	30.65	−54.32	−40.32
Egypt	F	2.17 (1.21–2.88)	3.95 (3.63–4.33)	1.83 (1.20–3.22)	82.03	−53.67	−15.67	0.46 (0.02–0.06)	0.07 (0.07–0.08)	0.03 (0.01–0.05)	−84.78	−57.14	−93.48
	M	4.50 (2.58–5.71)	4.93 (3.99–6.36)	3.48 (2.48–4.97)	9.56	−29.41	−22.67	0.10 (0.05–0.13)	0.10 (0.08–0.14)	0.06 (0.04–0.09)	0.00	−40.00	−40.00
Iran	F	0.30 (0.23–0.36)	1.84 (1.62–2.11)	5.24 (4.28–6.37)	513.33	184.78	1646.67	0.00 (0.00–0.00)	0.04 (0.03–0.04)	0.11 (0.09–0.14)	566.67	175.00	1733.33
	M	1.04 (0.81–1.30)	4.69 (4.05–5.49)	6.38 (5.37–7.56)	350.96	36.03	513.46	0.02 (0.01–0.02)	0.09 (0.08–0.14)	0.13 (0.11–0.15)	350.00	44.44	550.00
Iraq	F	1.20 (0.77–1.84)	6.34 (5.61–7.22)	8.64 (6.45–10.92)	428.33	36.28	620.00	0.02 (0.01–0.03)	0.12 (0.10–0.13)	0.16 (0.12–0.20)	500.00	33.33	700.00
	M	0.97 (0.61–1.50)	5.13 (4.55–5.93)	6.60 (4.99–8.03)	428.87	28.65	580.41	0.01 (0.01–0.03)	0.10 (0.09–0.11)	0.12 (0.09–0.15)	900.00	20.00	1100.00
Jordan	F	1.95 (1.49–2.32)	5.59 (5.01–6.36)	4.46 (3.74–5.41)	186.67	−20.21	128.72	0.03 (0.02–0.04)	0.11 (0.10–0.13)	0.09 (0.07–0.11)	266.67	−18.18	200.00
	M	1.11 (0.87–1.30)	4.85 (4.23–5.73)	8.64 (4.66–12.93)	336.94	78.14	678.38	0.02 (0.01–0.02)	0.10 (0.08–0.12)	0.19 (0.08–0.00)	400.00	90.00	850.00
Kuwait	F	4.49 (3.74–5.37)	7.52 (6.19–9.09)	3.54 (2.96–4.22)	67.48	−52.93	−21.16	0.08 (0.07–0.10)	0.14 (0.11–0.17)	0.06 (0.05–0.07)	75.00	−57.14	−25.00
	M	8.24 (7.46–9.15)	3.70 (3.35–4.12)	3.32 (2.95–3.74)	−55.10	−10.27	−59.71	0.17 (0.16–0.19)	0.06 (0.06–0.07)	0.06 (0.05–0.06)	−64.71	0.00	−64.71
Lebanon	F	26.98 (1.21–81.92)	33.72 (1.64–145.53)	33.53 (1.26–141.93)	24.98	−0.56	24.28	0.55 (0.01–1.71)	0.69 (0.01–2.95)	0.67 (0.01–2.88)	25.45	−2.90	21.82
	M	78.64 (7.93–246.37)	61.28 (5.07–293.73)	46.64 (2.96–300.57)	−22.08	−23.89	−40.69	1.83 (0.11–6.22)	1.37 (0.06–6.56)	1.04 (0.03–7.27)	− 25.14	−24.09	−43.17
Libya	F	5.61 (0.37–19.01)	30.88 (3.34–118.51)	44.74 (5.00–172.87)	450.45	44.88	697.50	0.11 (0.00–0.39)	0.63 (0.05–2.34)	0.89 (0.07–3.57)	472.73	41.27	709.09
	M	7.57 (0.94–25.79)	34.62 (4.52–141.72)	48.93 (5.47–229.06)	357.33	41.33	546.37	0.17 (0.01–0.66)	0.79 (0.06–3.34)	1.10 (0.08–5.68)	364.71	39.24	547.06
Morocco	F	10.23 (0.73–32.01)	72.07 (4.23–331.16)	42.55 (2.80–261.46)	604.50	−40.96	315.93	0.20 (0.00–0.65)	1.44 (0.04–6.42)	0.79 (0.02–5.41)	620.00	−45.14	295.00
	M	15.02 (1.73–49.89)	85.52 (5.64–395.22)	60.98 (3.29–389.23)	469.37	−28.70	305.99	0.34 (1.22–0.02)	1.92 (0.07–9.79)	1.32 (0.02–9.74)	464.71	−31.25	288.24
Oman	F	4.96 (3.94–5.84)	28.60 (21.17–36.02)	30.20 (16.39–49.88)	476.61	5.59	508.87	0.09 (0.07–0.11)	0.54 (0.39–0.70)	0.59 (0.29–1.04)	500.00	9.26	555.56
	M	13.41 (11.05–15.26)	79.30 (60.65–98.76)	154.21 (81.48–220.32)	491.35	94.46	1049.96	0.32 (0.26–0.36)	1.75 (1.28–2.25)	3.83 (1.96–5.46)	446.88	118.86	1096.88
Palestine	F	0.32 (0.25–0.39)	4.66 (4.08–5.21)	6.99 (6.11–8.05)	1356.25	50.00	2084.38	0.00 (0.00–0.00)	0.08 (0.07–0.09)	0.13 (0.11–0.15)	1233.33	62.50	2066.67
	M	2.57 (1.92–3.16)	8.19 (7.53–8.81)	8.59 (7.58–9.90)	218.68	4.88	234.24	0.05 (0.03–0.06)	0.16 (0.15–0.17)	0.17 (0.15–0.19)	220.00	6.25	240.00
Qatar	F	17.42 (12.23–21.54)	12.13 (10.01–15.56)	9.87 (7.05–12.06)	−30.37	−18.63	−43.34	0.33 (0.23–0.41)	0.23 (0.18–0.31)	0.19 (0.14–0.23)	−30.30	−17.39	− 42.42
	M	16.94 (12.44–20.79)	10.63 (9.03–12.27)	7.16 (6.25–8.48)	−37.25	−32.64	−57.73	0.35 (0.26–0.43)	0.23 (0.19–0.28)	0.14 (0.13–0.17)	−34.29	−39.13	−60.00
Saudi Arabia	F	14.08 (9.25–20.89)	53.30 (47.42–59.74)	53.20 (41.09–67.90)	278.55	−0.19	277.84	0.29 (0.18–0.43)	1.11 (1.00–1.23)	1.10 (0.83–1.45)	282.76	−0.90	279.31
	M	15.99 (10.55–23.74)	45.84 (40.60–52.34)	41.29 (31.24–49.96)	186.68	−9.93	158.22	0.36 (0.23–0.56)	1.05 (0.91–1.19)	0.90 (0.65–1.13)	191.67	−14.29	150.00
Sudan	F	27.35 (17.73–50.25)	608.88 (420.73–848.27)	688.55 (929.31–929.31)	2126.25	13.08	2417.55	0.50 (0.23–0.97)	12.60 (8.75–17.56)	14.36 (10.13–19.15)	2420.00	13.97	2772.00
	M	58.40 (37.05–115.57)	513.81 (356.86–736.59)	479.49 (339.26–698.76)	779.81	−6.68	721.04	1.17 (0.71–2.43)	11.12 (7.89–16.15)	10.29 (7.33–14.97)	850.43	−7.46	779.49
Syria	F	1.67 (1.38–1.92)	3.41 (2.82–3.88)	3.13 (2.30–4.52)	104.19	−8.21	87.43	0.03 (0.02–0.03)	0.06 (0.05–0.07)	0.05 (0.03–0.08)	100.00	−16.67	66.67
	M	1.81 (1.59–2.03)	3.18 (2.39–3.72)	2.30 (1.48–4.19)	75.69	−27.67	27.07	0.03 (0.04–0.03)	0.06 (0.04–0.07)	0.03 (0.02–0.07)	100.00	−50.00	0.00
Tunisia	F	2.12 (0.47–11.00)	21.47 (2.99–107.87)	49.23 (3.13–216.30)	912.74	129.30	2222.17	0.04 (0.00–0.22)	0.41 (0.03–2.13)	0.94 (0.03–4.44)	925.00	129.27	2250.00
	M	6.13 (1.03–29.39)	31.56 (3.33–132.56)	52.52 (3.55–177.10)	414.85	66.41	756.77	0.13 (0.01–0.69)	0.70 (0.02–3.39)	1.15 (0.03–4.27)	438.46	64.29	784.62
Turkey	F	0.25 (0.01–0.47)	2.32 (1.85–3.12)	6.01 (4.78–7.07)	828.00	159.05	2304.00	0.00 (0.00–0.00)	0.04 (0.03–0.06)	0.11 (0.09–0.14)	700.00	175.00	2100.00
	M	0.67 (0.03–1.27)	6.2 (4.80–8.59)	11.15 (8.73–13.30)	825.37	79.84	1564.18	0.01 (0.00–0.02)	0.13 (0.09–0.19)	0.24 (0.18–0.29)	1200.00	84.62	2300.00
United Arab Emirates	F	3.04 (0.13–10.27)	19.78 (2.12–76.82)	25.69 (2.84–103.71)	550.66	29.88	745.07	0.06 (0.00–0.21)	0.40 (0.03–1.55)	0.52 (0.04–2.09)	566.67	30.00	766.67
	M	6.21 (0.64–21.75)	36.42 (4.75–143.26)	228.95 (5.97–1483.06)	486.47	528.64	3586.80	0.14 (0.00–0.53)	0.81 (0.06–3.40)	10.58 (0.10–74.53)	478.57	1206.17	7457.14
Yemen	F	13.40 (0.66–59.01)	31.20 (2.75–115.35)	29.22 (1.35–152.51)	132.84	−6.35	118.06	0.27 (0.00–1.30)	0.63 (0.04–2.38)	0.55 (0.01–2.99)	133.33	−12.70	103.70
	M	41.17 (2.87–198.04)	75.53 (8.92–284.29)	75.81 (3.46–445.03)	83.46	0.37	84.14	0.94 (0.03–4.69)	1.72 (0.14–6.63)	1.63 (0.03–10.45)	82.98	−5.23	73.40
Middle East and North Africa	**F**	**5.14 (3.34–8.07)**	**47.58 (33.63–69.06)**	**53.44 (38.79–75.89)**	**825.68**	**12.32**	**939.69**	**0.10 (0.06–0.16)**	**0.95 (0.67–1.39)**	**1.05 (0.77–1.50)**	**850.00**	**10.53**	**950.00**
	**M**	**10.06 (6.61–16.18)**	**45.87 (32.10–70.37)**	**46.16 (31.30–72.66)**	**355.96**	**0.63**	**358.85**	**0.22 (0.13–0.37)**	**1.00 (0.69–1.58)**	**1.00 (0.65–1.63)**	**354.55**	**0.00**	**354.55**

## Results

The rate of DALYs/100,000 attributed to drug use for AIDS and percentage changes from 1990 to 2017 in MENA countries are shown in Table 1. In MENA countries, the rate of DALYs/100,000 attributed to drug use for AIDS in women, 1.10 (95% CI: 0.75–1.71) in 1990, 10.60 (95% CI: 10.6–15.27) in 2007 and 13.39 (95% CI: 9.98–18.17) in 2017. In men, there is an increasing trend from 1990 to 2017. The rate of DALYs/100,000 attributed to drug use for AIDS in men living in United Arab Emirates, and women living in Tanzania and Sudanese increased from 2007 to 2017, and these countries had the highest percentage of change.

In Iran, rate of DALYs/100,000 attributed to drug use for AIDS in men (27.53 to 33.52) and women (8.47 to 20.62) increased from 2007 to 2017, with percent change of 21.76 and 143.45% in men and women, respectively. The rate of DALYs/100,000 attributed to drug use for AIDS decreased from 2007 to 2017 in the Bahrain, Lebanon, Egypt, Morocco and Qatar.

**Fig. 1:**
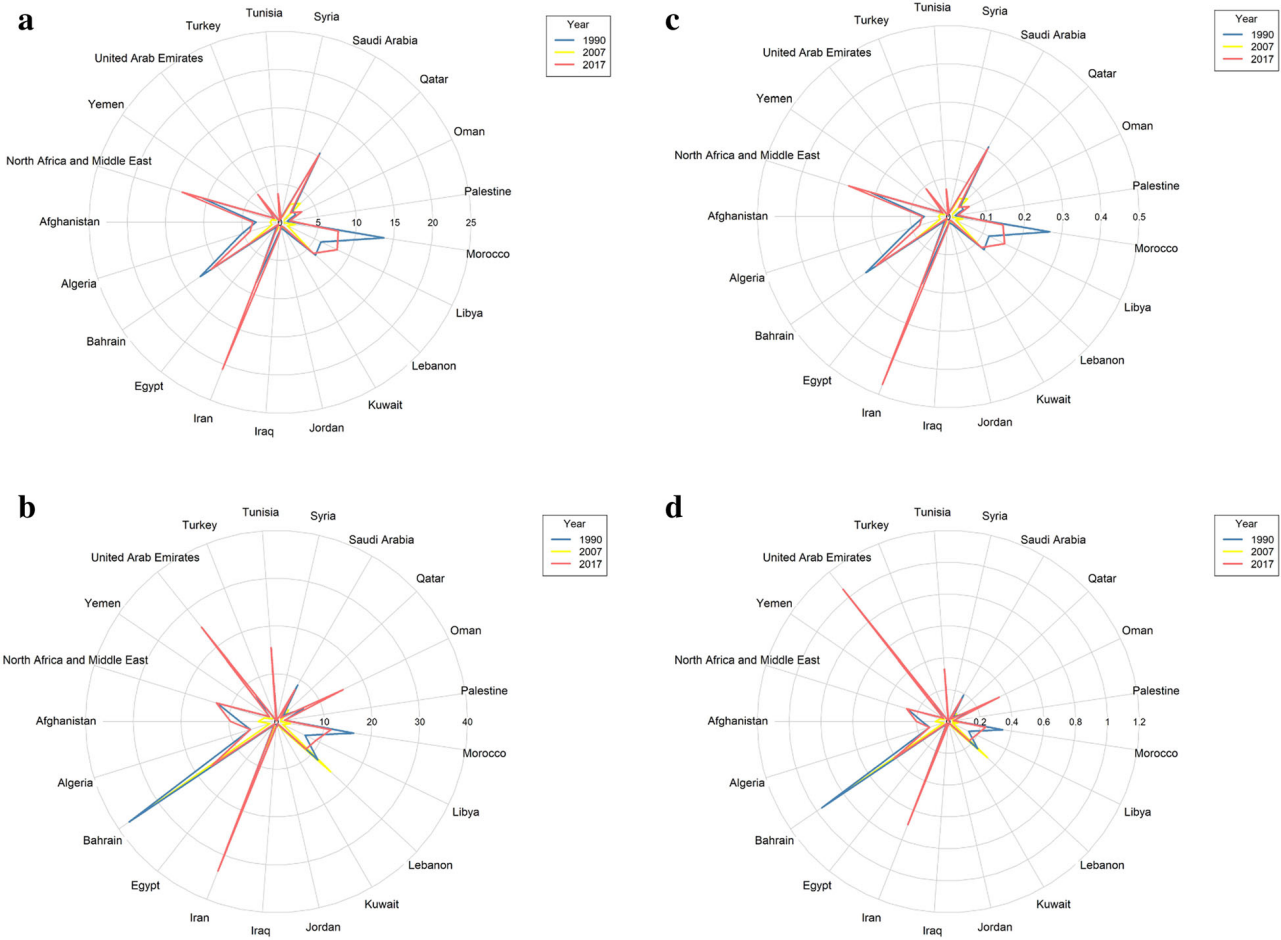
Trend of the attributable mortality and burden of drug use in AIDS at MENA countries, **a** DALY in Women, **b** DALY in Men, **c** Age Standardized Mortality Rate in Women, **d** Age Standardized Mortality Rate in Men

**Fig. 2:**
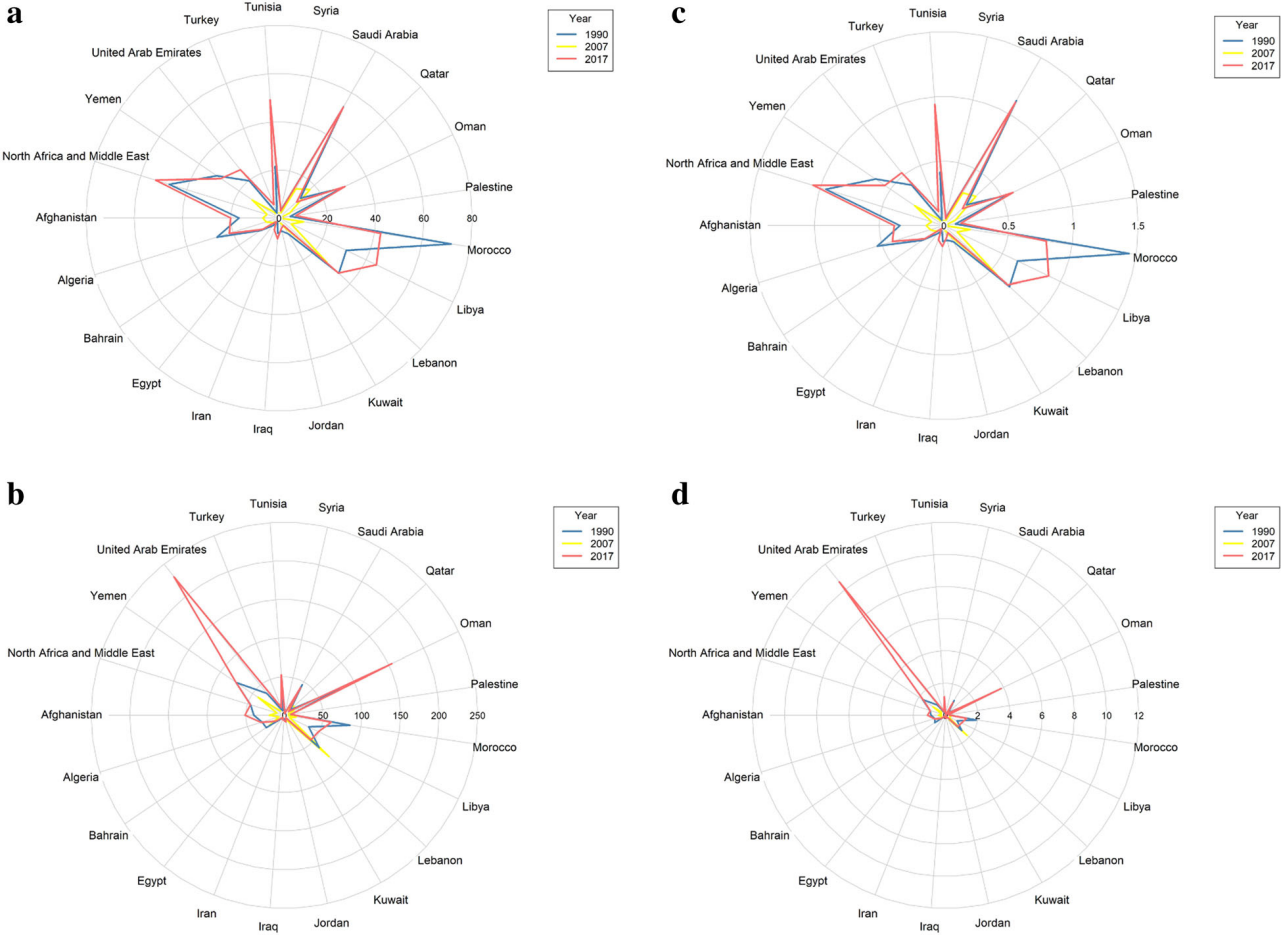
Trend of the attributable mortality and burden of unsafe sex in AIDS at MENA countries, **a** DALY in Women, **b** DALY in Men, **c** Age Standardized Mortality Rate in Women, **d** Age Standardized Mortality Rate in Men

In most MENA countries, the rate of DALYs/100,000 and age-standardized mortality rate attributed to unsafe sex and drug use for AIDS has increased in men and women (Figs. 1 and 2).

The rate of DALYs/100,000 attributed to unsafe sex for AIDS and percentage changes in MENA countries from 1990 to 2017 are shown in Table 2. In some countries, such as Afghanistan, Bahrain, Kuwait, Egypt and Qatar attributable DALYs to unsafe sex increased 1990 to 2007 and then decreased in 2017. Age-standardized mortality rate has also shown a state of DALYs.

In all countries of the MENA, rate of DALYs/100,000 attributed to unsafe sex for AIDS is higher in men than in women except Qatar, Saudi Arabia and Sudan. Age-standardized mortality rate attributed to unsafe sex for AIDS is higher in men than in women except Saudi Arabia and Sudan.

In women of MENA countries, the rate of DALYs/100,000 attributed to unsafe sex for AIDS in 1990, 2007 and 2017 were 5.14 (3.34–8.07), 47.58 (33.63–69.06) and 53.44 (38.79–75.89), respectively; in men 10.06 (6.61–16.18), 45.87 (32.10–70.37) and 46.16 (31.30–72.66).

Age-standardized mortality rate in 1990, 2007 and 2017 were 0.10 (0.06–0.16), 0.95 (0.67–1.39) and 1.05 (0.77–1.50) for women and 0.22 (0.13–0.37), 1.00 (0.69–1.58) and 1.00 (0.65–1.63) in men of MENA countries.

Age-standardized mortality rate attributed to unsafe sex for AIDS increased by 550% in men and 11,733% in women; attributable DALYs/100,000 due to unsafe sex increased by 350% in men and 513% in women from 1990 to 2017 in Iran.

**Fig. 3:**
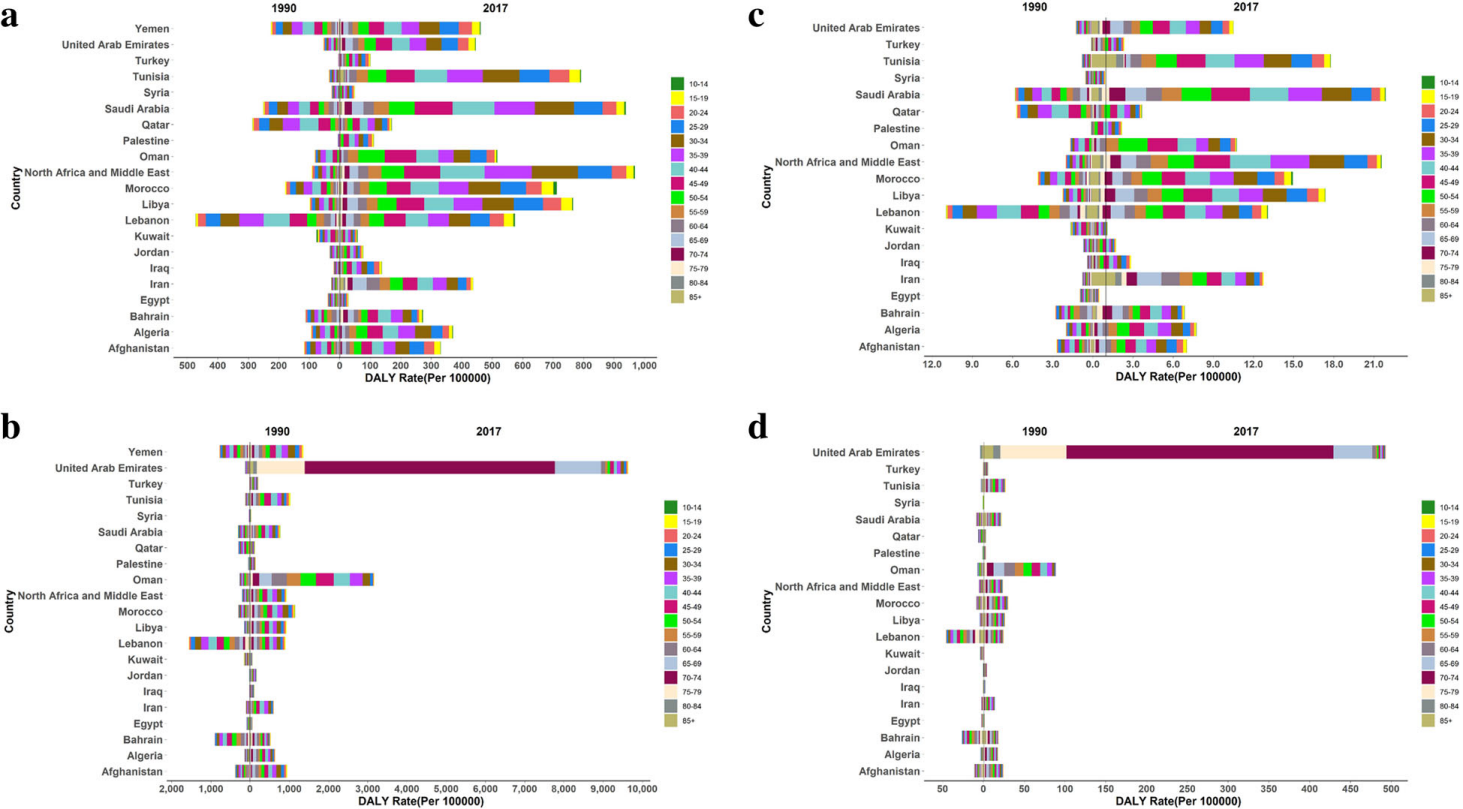
Age trend of the attributable mortality and burden of drug use in AIDS at MENA countries, **a** DALY in Women, **b** DALY in Men, **c** Age Standardized Mortality Rate in Women, **d** Age Standardized Mortality Rate in Men

**Fig. 4:**
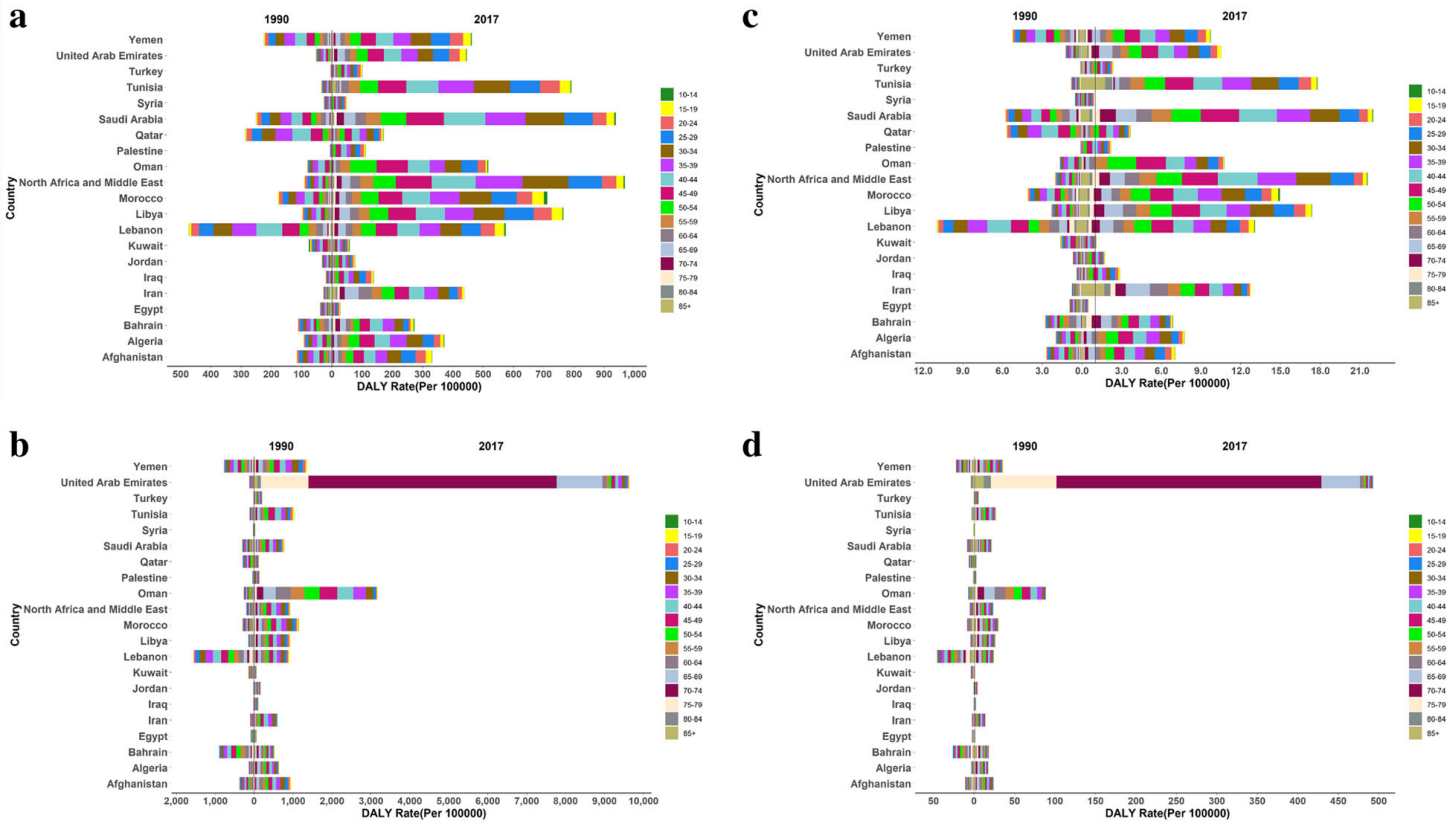
Age trend of the attributable mortality and burden of unsafe sex in AIDS at MENA countries, **a** DALY in Women, **b** DALY in Men, **c** Age Standardized Mortality Rate in Women, **d** Age Standardized Mortality Rate in Men

Age trend of the mortality and burden of drug use and unsafe sex in AIDS at MENA countries both of sex are shown in Figs. 3 and 4. The rate of DALYs/100,000 of the two risk factors (unsafe sex and drug use) in Sudan was much higher than in other countries; thus, it is not shown in Figs. 3 and 4. In women less than 65 years, the rate of DALYs/100,000 attributed to drug use for AIDS increased in the Morocco and Saudi Arabia from 1990 to 2017, this increase has been lower in men than women. The rate of DALYs/100,000 attributed to drug use for AIDS no significant change in Syria and Egypt in all age groups (Fig. 3a and b). Age-standardized mortality rate attributed to drug use for AIDS increased in United Arab Emirates, Tunisia, Iran and Libya in all age groups of women (Fig. 3c).

In all age groups of women, the rate of attributable burden of unsafe sex in AIDS increased from 1990 to 2017 at MENA countries, especially in United Arab Emirates, Tunisia, Libya, Morocco, Saudi Arabia and Iran (Fig. 4a). Age-standardized mortality rate attributed to unsafe sex in AIDS increased from 1990 to 2017 at MENA countries, especially in United Arab Emirates, Oman, Tunisia, Libya, Morocco, Saudi Arabia and Iran in all age groups of women (Fig. 4c).

## Discussion

The findings of this study showed the rate of DALYs/100,000 and age-standardized mortality rate attributed to risk factors (drug use and unsafe sex) underwent dramatic changes across the MENA countries over the period 1990 to 2017. The rate of DALYs/100,000 and age-standardized mortality rate attributed to drug use for AIDS increased during the study period in men and women. The rate of DALYs/100,000 and age-standardized mortality rate attributed to drug use and unsafe sex for AIDS increased in the MENA countries in age groups less than 65 years from 1990 to 2017, the increase in women more than men.

According to the report from world health organization (WHO), this region has the first rank in the speed of growing epidemic of HIV/AIDS in the world [13]. The most changes occurred in men of the United Arab Emirates, Tanzanian men and Sudanese women. The rate of DALYs/100,000 attributable to these two risk factors (unsafe sex and drug use) in Sudan was much higher than in other countries.

In the study of Degenhardt L et al. (2013) more than 10 million DALYs was estimated to be attributable to previous exposure to HIV, HBV, and HCV via IDU [7]. Study by Singh K et al. (2019) has shown PWID are at a disproportionately increased risk of death due to overdose and suicide [20]. However, the increased risk of death can be related to other causes associated with intravenous drug abuse rather than HIV [21]. Therefore, determination of the cause of death in such individuals is important and somewhat difficult.

Our findings showed the rate of DALYs/100,000 and age-standardized mortality rate attributed to unsafe sex for AIDS increased in MENA from 1990 to 2017. In some countries, such as Afghanistan, Bahrain, Kuwait, Egypt and Qatar DALYs attributable to unsafe sex increased 1990 to 2007 and then decreased in 2017. In all countries of the MENA, DALYs/100,000 and mortality rate attributed to unsafe sex in men is more than women.

In most countries, HIV epidemics often spread initially among key populations like PWID or Women sex workers (FSWs) and men who have sex with men (MSM). FSWs usually have high numbers of sexual partners and transmit the disease to other key populations (PWID and pimps) and to their clients [22]. Though FSWs have been shown to use condoms more often in commercial than in private sexual contacts [22], it should be noted that unprotected sex involving FSWs remains common in certain regions [23]. Fifteen percent of the global HIV burden is due to unsafe sex in women who have chosen sex with multiple men as a job, resulting in more than 100,000 deaths per year [19]. In Cotonou, the capital of Benin, it was assumed that nearly all HIV infections in women and 76% in men were due to sexual contacts with FSWs [24, 25]. The rapid and early spread of HIV in some Asian countries like Cambodia and Thailand has been associated with a high use of commercial sex [26]. A Indian nationally survey found that about 4% of Indian men visited a FSW in the previous year with much higher percentages in regions with high HIV prevalence [27]. Women sex work is an important factor to HIV transmission and the global HIV burden [19].

A study in South Africa, drinking alcohol has introduced as a mediator variable between unsafe sex and AIDS. Evidence has shown that drinking alcohol independently affects people’s sexual performance, and undermines skills for condom negotiation and correct use [28]. A study using structural-environmental model has shown association between alcohol and the risk of HIV/AIDS by sexual contact in Latino Migrant Day Laborers; as discrimination and working conditions worsen, contact with family decreases, drinking becomes more problematic, and sexual risk increases [29]. The association between alcohol and unsafe sex has been observed among American college students [30]. A meta-analysis study has reported, people who consume alcohol had significantly multiple partners, sex without a condom or inconsistent use of condoms [31].

Suárez-García I et al., reported patients who acquired HIV by sexual transmission compared with who acquired HIV infection through use of injected drugs has higher risk of late presentation, delayed antiretroviral therapy (ART) initiation, higher mortality and risk of progression to AIDS [32]. Therefore, in examining the risk factors for AIDS, it is also important to pay attention to the mediator factors; which can reduce or increase DALYs and mortality rate attributed to AIDS.

This study was conducted using data GBD; therefore, such estimate might be different from the real scenario where there is no information for some low-income countries. However, use of exact and modern statistical methods provides the best estimate for all included countries. For conditions such as drug use and unsafe sex in MENA countries where there is more sever social stigma and legal ban, the confidence about such data might be under more question. However, our main objective was to present the DALY and mortality rate attributed to unsafe sex and drug use for AIDS which can provide valid result of burden of unsafe sex and drug use in AIDS for each year and both sex in MENA countries.

## Conclusion

Given the rapid increase in the burden of HIV/AIDS related to unsafe sex and drug use in all age groups in women and men at MENA countries, with recent political instability in some countries in this region in addition to lower available resources because of economical constrain dur to epidemic of COVID-19, one can expect to observe the greater burden related to these two risk factors in near future in all the world and MENA countries. Therefore, it is quite necessary to address the size of real psychosocial burden of drug use and unsafe sex for all policy makers to provide, continue and improve the previous public health plans related to HIV/AIDS in MENA.

## Data Availability

The datasets used and analyzed during the current study are available from the corresponding author on reasonable request.

## Abbreviations

AIDS: Acquired immunodeficiency syndrome
DALYs: Disability adjusted life years
GBD: Global burden of disease
HIV: Human immunodeficiency virus
HBV: Hepatit B virus
HCV: Hepatit C virus
IDU: Injecting drug use
IHME: Institute for Health Metrics and Evaluation
MENA: Middle East and North Africa
PWID: Including people who inject drugs
SWs: Sex workers
WHO: World health organization
