# Correction to: Disability-adjusted life years and mortality rate attributed to unsafe sex and drug use for AIDS in the Middle East and North Africa countries

**DOI:** 10.1186/s13690-021-00527-1

**Published:** 2021-01-18

**Authors:** Farid Najafi, Fatemeh Khosravi Shadmani, Mojtaba Ghalandari, Mitra Darbandi

**Affiliations:** 1 Research Center for Environmental Determinants of Health (RCEDH), Health Institute, Kermanshah University of Medical Sciences, Kermanshah, Iran; 2 Cardiovascular Research Center, Kermanshah University of Medical Sciences, Kermanshah, Iran; 3 Mahneshan Health Center, Zanjan University of Medical Sciences, Zanjan, Iran; 4 Department Epidemiology, Public Health College, Kermanshah University of Medical Sciences, Kermanshah, Iran

**Correction to: Arch Public Health 78, 130 (2020)**

**https://doi.org/10.1186/s13690-020-00511-1**

Following publication of the original article [1], the authors identified an error in the author name of Fatemeh Khosravi Shadmani

- The incorrect author name is: Fatemeh Khosravi
- The correct author name is: Fatemeh Khosravi Shadmani

The author group has been updated above and the original article [1] has been corrected.
